# The Effect of Glucagon-like-Peptide-1 Receptor Agonists on Diabetic Retinopathy Progression, Central Subfield Thickness, and Response to Intravitreal Injections †

**DOI:** 10.3390/jcm13206269

**Published:** 2024-10-21

**Authors:** Tomer Michaeli, Samer Khateb, Jaime Levy

**Affiliations:** 1 “Tzameret”, Faculty of Medicine, The Hebrew University of Jerusalem, Jerusalem 91120, Israel; tomer.michaeli1@mail.huji.ac.il; 2 Medical Corps, Israel Defense Forces, Ramat Gan 52625, Israel; 3 Division of Ophthalmology, Hadassah Medical Center, Faculty of Medicine, The Hebrew University of Jerusalem, Jerusalem 91120, Israel; samer.khateb@mail.huji.ac.il

**Keywords:** diabetic retinopathy, glucagon-like-peptide-1 receptor agonists (GLP1-RA), central macular edema, anti-VEGF intravitreal injections

## Abstract

**Objectives:** To examine the effects of glucagon-like-peptide-1 receptor agonists (GLP1-RAs) on diabetic retinopathy (DR) progression, visual acuity (VA), central subfield thickness (CST), and response to intravitreal injections (IVIs) in the Hadassah ophthalmological cohort. **Methods:** Of 4500 Hadassah patients with DR, 146 had a documented first course of GLP1-RA treatment lasting at least a year along with ophthalmological follow-up. Of these, 35 underwent at least two optical coherence tomography (OCT) exams with a one-year interval. These 35 GLP1-RA–naïve patients were compared to a control group of 31 patients with DR who did not receive GLP1-RA treatment. We compared demographics, medical records, ocular data, and OCT characteristics between the two study groups. **Results:** At baseline, patients who received GLP1-RA treatment had a significantly higher prevalence of retinal detachment and vitreous hemorrhage, as well as a higher (though not statistically significant) prevalence of cardiovascular comorbidities compared to the control group. At the end of the follow-up period, the GLP1-RA group had a higher prevalence of DR progression compared to controls (3/19 vs. 0/20, respectively; *p* = 0.106, Fisher’s exact test), but also showed a better response to IVIs (27/35 vs. 17/31, respectively; unadjusted OR: 2.78, *p* = 0.058; 95% CI: [0.963, 8.020], Pearson’s chi-square test). However, vitreous hemorrhage and hyperreflective retinal foci were confounding factors (adjusted IVI response OR: 1.76, *p* = 0.229, 95% CI: [0.553, 5.650], logistic regression). No significant differences were observed between the two groups in terms of change in visual acuity (−0.135 vs. −0.063 logMAR, respectively; *p* = 0.664, Student’s *t*-test) or CST (−13.49 vs. −30.13 μm; *p* = 0.464, Student’s *t*-test). **Conclusions:** This study presents preliminary findings showing no significant differences in DR progression, visual acuity, and CST between patients treated with GLP1-RA and control patients. Moreover, GLP1-RA therapy was not significantly associated with improved IVI response, with ocular parameters acting as confounding factors.

## 1. Introduction

Diabetic retinopathy (DR) is a leading cause of preventable vision loss, affecting nearly a third of patients with diabetes mellitus [1]. Current treatments typically involve intravitreal injections (IVIs) and laser therapy to manage complications [1,2].

Glucagon-like peptide-1 receptor agonists (GLP1-RAs) are a promising class of drugs for type 2 diabetes mellitus (T2DM). These drugs improve blood sugar control by mimicking the effects of incretins [3]. While establishing good glycemic control can reduce the long-term risk of DR, some studies suggest a potential short-term worsening of DR with rapid blood sugar reduction [4,5].

Beyond their effect on glycemic control, GLP1-RAs have shown evidence of several other retinal protective mechanisms. Studies by Pang B et al. 2017 [6] and Wei L et al. 2022 [7] demonstrated the protective effect on the retinal blood barrier in mouse models. Additionally, He X et al. 2024 [8] revealed that GLP1-Ras protects against DR pathogenesis in retinal endothelial cells, diabetic mice, and patients by inhibiting the interferon gene pathway (STING), which regulates innate immune responses and vascular diseases [8].

However, clinical studies regarding the effects of GLP1-RAs on DR progression and ocular complications have yielded inconclusive results [9]. Several studies have found a decreased but not statistically significant incidence of ocular complications and reduced risk of DR progression in patients receiving GLP1-RA therapy [10,11,12]. For example, the HARMONY trial demonstrated a reduced rate of ocular complications (OR: 0.88, 95% CI: [0.64, 1.19], *p* = 0.392) [10]. In contrast, other studies [13,14,15], such as the SUSTEIN-6 trial, observed an increased risk (OR: 1.75, 95% CI: [1.10, 2.78], *p* = 0.018) [13]. These conflicting results were reflected in a meta-analysis using a random-effects model, which found no association between GLP1-RA treatment and the risk of DR progression [9].

These differences in results may be explained—at least in part—by the limitations of the studies, as they had a relatively generalized design and measured ocular data only as a secondary cardiovascular outcome. Additionally, most of these studies lacked detailed eye examinations [11,12,14,15,16].

Given these complexities, further research is needed to clarify the putative relationship between GLP1-RA treatment and DR progression. Importantly, studies to date have not analyzed the association between GLP1-RA treatment and changes in subsequent DR treatment protocols, nor have they specifically examined optical coherence tomography (OCT) scans alongside DR progression. The FOCUS trial—the results of which are expected in 2027 or later—is designed to address these gaps by combining detailed ophthalmic examinations with an assessment of DR progression and associated treatments [17].

In the present article, we examined both DR outcomes and OCT scans, alongside DR treatments, in the Hadassah ophthalmological cohort. We compared patients who received their first course of GLP1-RA treatment to a control group of patients who did not receive GLP1-RAs. Importantly, this study incorporated a comprehensive combination of clinically relevant and ocular parameters that had not previously been combined in a single study, thus yielding novel insights.

## 2. Methods

### 2.1. Study Design

The study design was a medical record–based retrospective analytic study that included both control and in-group comparisons between patients who received their first GLP1-RA treatment course and control patients who did not receive GLP1-RAs. The main outcomes were the rates of DR progression, DR complications, and the rate and response to DR-associated treatments, including anti-VEGF IVIs.

### 2.2. The Data

**Figure 1:**
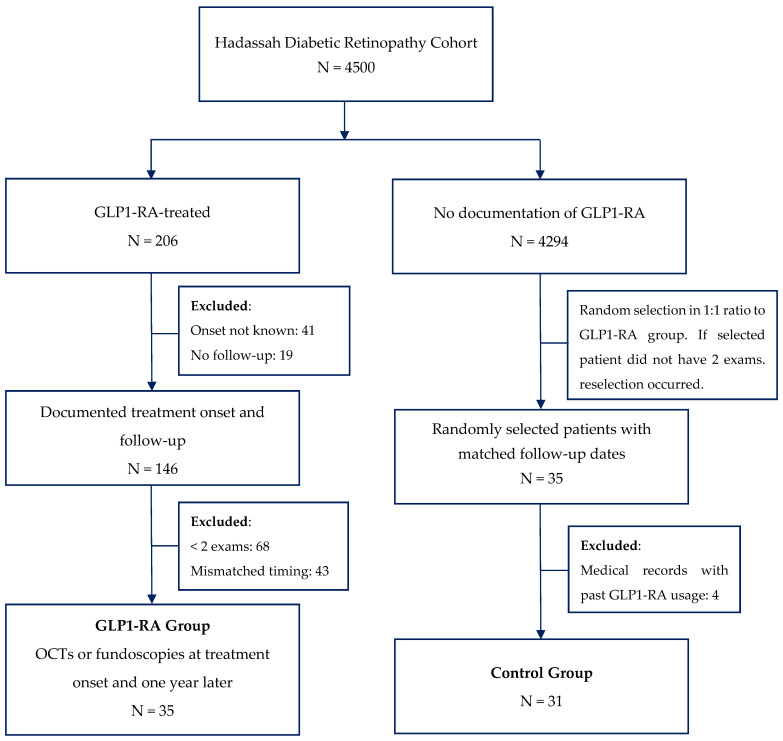
Flowchart of the patient selection process. Note the large drop in patient numbers in the first step and the final exclusion of 4 patients from the control group due to the finding that these patients received GLP1-RA treatment in the past, revealed during a manual review of the medical records.

All data collection was approved by the Hadassah Institutional Ethics Committee (approval number 0382-19). We examined medical records and OCT images obtained at the Hadassah Ophthalmology Department, with no personally identifiable information. The selection process is depicted in Figure 1. Using Python version 3.11, we scanned 40,000 visits by 4500 patients with DR who received follow-up care at the Hadassah medical institution. Of these 4500 patients, 146 patients began a new course of GLP1-RA treatment during their follow-up care and were therefore GLP1-RA–naïve. Of these 146 patients, only 35 underwent an OCT exam and/or fundoscopy both at treatment onset and one year later. A control group was randomly selected, matched only by appointment dates.

### 2.3. Inclusion Criteria

We applied the following inclusion criteria: (i) a confirmed diagnosis of T2DM; (ii) a follow-up duration of at least one year before initiating treatment with GLP1-RAs and one year after starting treatment; (iii) for the GLP1-RA–treated group, at least one color fundus examination or OCT scan 0–6 months before initiating GLP1-RA treatment, and 1–1.5 years afterward; and (iv) for the control group, at least two-time points with color fundus examinations or OCT scans, with follow-up visits matching the time points for the patients in the GLP1-RA group, as well as no prior history of taking GLP1-RAs.

### 2.4. Study Groups

A full manual examination of the medical records of the aforementioned 146 patients in the GLP1-RA group revealed that only 35 patients met all of the inclusion criteria. Patients in the control group were randomly selected from the rest of the Hadassah cohort. After a comprehensive review of their medical records to ensure they met the inclusion criteria, these patients were matched only by their follow-up dates, not by any other variables or DR stage. To assess potential confounders, BMI (body mass index), HbA1c (hemoglobin A1c) levels, and DR stage were also compared between the two groups at the beginning of the follow-up period.

### 2.5. Measured Outcomes

#### 2.5.1. Primary Outcomes

The primary outcomes assessed were as follows: DR stage progression measured by color fundus examination or OCT scan, visual acuity (logMAR), and response to IVI therapy. DR progression was defined as an increase in the DR Disease Severity Scale by at least one level (mild non-proliferative diabetic retinopathy (NPDR), moderate NPDR, severe NPDR, proliferative diabetic retinopathy (PDR)) during a year of follow-up, as measured by color fundus examination or OCT scan [18,19].

Patients received IVIs following the “modified treat-and-extend protocol” [20]. Although the response to IVIs has not been extensively studied in patients with DR, evidence suggests that the parameters of the treat-and-extend protocol can reflect response to IVIs [21] and may vary across different clinical clusters of patients, as observed in neovascular age-related macular degeneration patients [22].

In the current study, the response to IVI therapy was defined as a categorial parameter, with the following values: “Good”, “Stable”, and “Poor”. A good response was defined as (i) an extension of at least 2 weeks in the injection interval over a year; (ii) no need for further IVIs at the end of the follow-up period. A poor response was defined as (i) a reduction of at least 2 weeks in the injection interval over a year; (ii) a switch in medication from bevacizumab to ranibizumab or aflibercept; and/or (iii) the initiation of new IVI anti-VEGF therapy during follow-up. A stable response was defined as patients who did not meet the criteria for either a good or poor response.

#### 2.5.2. Secondary Outcomes

OCT parameters were assessed as secondary outcomes. All OCT images were anonymized, and their association with the specific study group was masked. One of the senior authors (JL) interpreted the images, measuring central macular thickness and noting the presence of intraretinal fluid (IRF), subretinal fluid (SFR), exudates, and hemorrhages. Additionally, any ocular surgeries performed during the follow-up period to examine the patients’ IVI response were documented as a secondary outcome.

### 2.6. Measured Variables

#### 2.6.1. Categorial Variables

Baseline diabetes-mellitus-associated complications were included as categorical variables and consisted of diabetic nephropathy, diabetic neuropathy, cardiovascular comorbidities, and peripheral vascular disease. Other categorical variables included the use of medications at baseline, and a binary representation of various DR complications such as diabetic macular edema, vitreous hemorrhage, neovascular glaucoma, and retinal detachment. Finally, DR stage and treatments at baseline were also recorded as categorical variables, including anti-VEGF IVIs, laser therapy, and vitrectomy. Regarding IVIs, therapies in use were only anti-VEGF medications, including bevacizumab (Avastin, Genentech/Roche, CA, USA), ranibizumab (Lucentis, Genentech, CA, USA), and aflibercept (Eylea, Regeneron Pharmaceuticals, NY, USA). Shifts between treatment lines were also recorded for each eye.

#### 2.6.2. Quantitative Variables

Quantitative variables included the first documented BMI (kg/m^2^) and HbA1c (%) during follow-up, macular thickness (microns), the duration of T2DM and DR, and the interval between injections (weeks), and visual acuity (VA, logMAR).

### 2.7. Statistical Analyses

All statistical tests were two-sided, and differences with a *p*-value ≤ 0.05 were considered significant. The association between two dichotomous categorical variables was analyzed using Pearson’s chi-squared test or Fisher’s exact test. For non-dichotomous categorical variables, Fisher–Freeman–Halton’s exact test was used. Quantitative variables between the two groups were compared using Student’s *t*-test or the Mann–Whitney *U* test. Quantitative variables between more than two groups were compared using the Kruskal–Wallis test. The association between two quantitative variables was analyzed using Pearson’s correlation coefficient. A dichotomous dependent multivariable model was analyzed using logistic regression. The significance of associations between features in binary classification was assessed using Fisher’s exact test. Quantitative-dependent multivariable models were analyzed using regression models. Categorical variables are presented as the number of patients (N) and percentage (%), while quantitative variables are presented as the mean ± standard deviation (SD).

## 3. Results

### 3.1. Between-Group Descriptive Analysis

The statistically significant differences between patients in the GLP1-RA and the control group were as follows: follow-up duration (598 ± 449 vs. 404 ± 117 days, respectively; *p* = 0.017, Student’s *t*-test), prevalence of vitreous hemorrhage at baseline (45.7% vs. 19.4%, respectively; *p* = 0.023, Pearson’s chi-square), a previous history of IVIs (60.0% vs. 90.0%, respectively; *p* = 0.005, Pearson’s chi-square), baseline central subfield thickness (CST; 305.54 ± 76.25 vs. 362.55 ± 115.24 microns, respectively; *p* = 0.02, Student’s *t*-test), and hyperreflective retinal foci (HRF; 11.4% vs. 35.5%, respectively; *p* = 0.02, Pearson’s chi-square). Other notable variables that differed between groups but did not reach statistical significance were: tobacco use (37.1% vs. 19.4%, respectively; *p* = 0.11, Pearson’s chi-square), insulin use (82.9% vs. 64.5%, respectively; *p* = 0.09, Pearson’s chi-square), SGLT2 inhibitor use (42.9% vs. 25.8%, respectively; *p* = 0.15, Pearson’s chi-square), vitrectomy at baseline (20.0% vs. 6.5%, respectively; *p* = 0.10, Pearson’s chi-square), and presence of IRF (62.9% vs. 80.6%, respectively; *p* = 0.11, Pearson’s chi-square). A summary of baseline variables is provided in Table 1.

### 3.2. Ocular Effects of GLP1-RA Treatment

None of the primary outcomes differed significantly between the two study groups. Although DR progression was not significantly associated with the GLP1-RA group compared to the control group (3/19 vs. 0/20, respectively; *p* = 0.106, Fisher’s exact test), the incidence was low after one year of follow-up. However, a “Good” response (see Methods) to anti-VEGF IVIs tended to be associated with the GLP1-RA group compared to the control group (27/35 vs. 17/31, respectively; *p* = 0.055, Pearson’s chi-square). No difference was found between the GLP1-RA and control groups concerning the change in VA (−0.135 vs. −0.063 logMAR, respectively; *p* = 0.664, Student’s *t*-test) or the change in CST (−13.49 vs. −30.13, respectively; *p* = 0.464, Student’s *t*-test). During follow-up, new vitrectomies and laser therapies were documented, with no statistically significant differences between study groups. The various outcomes of the two study groups are summarized in Table 2.

### 3.3. DR Progression

To calculate DR progression, 16 and 11 patients in the GLP1-RA and control groups, respectively, with a baseline DR severity of PDR were excluded, resulting in 19 GLP1-RA-treated patients and 20 patients in the control group with a DR severity between mild and severe NPDR. Of these patients, 3/19 (15.8%) and 0/20 (0%) in the GLP1-RA and control groups, respectively, had an increase in severity after follow-up (*p* = 0.106, Fisher’s exact test). However, the limited number of DR progression cases reduces the power to detect significant trends (i.e., 3 and 0 patients in the GLP1-RA and control groups, respectively). Between-group comparisons of laser therapy and vitrectomies performed at baseline also revealed no significant differences. More details are provided in Table S3 in the Supplementary Material.

### 3.4. Visual Acuity

Although a change in VA of at least 2 lines (or ±0.2 LogMAR units) is commonly considered significant [23], the difference in the mean change in VA between the study groups was only 0.072 LogMAR; moreover, both the GLP1-RA and control groups had a change that was lower than this threshold (−0.135 ± 0.133 vs. −0.063 ± 0.092 LogMAR, respectively; *p* = 0.664, Student’s *t*-test). Regarding the change in VA relative to the baseline variables, the only statistically significant association was found between poor VA at baseline and higher improvement in VA during follow-up (Pearson’s correlation coefficient: −0.721, 95% CI: [−0.820, −0.580], *p* < 0.001), although baseline laser therapy was slightly—albeit not significantly—associated with higher improvement in VA, with patients receiving laser therapy having a VA change of −0.25 ± 0.13 LogMAR compared to a change of 0.04 ± 0.09 LogMAR in patients who did not receive laser therapy (*p* = 0.078, Pearson’s chi-square). A multivariable linear regression model was also generated, yielding an adjusted R^2^ value of 0.569; thus, GLP1-RA treatment was not identified as a statistically significant variable. In addition, variables were selected via a stepwise selection process applied to all categories of baseline characteristics, with the use of GLP1-RAs as a fixed variable. The selected variables were as follows: VA at baseline (B = −0.865, *p* < 0.001, 95% CI: [−1.056, −0.675]), duration of T2DM (B = 0.017, *p* = 0.019, 95% CI: [0.03, 0.030]), retinal detachment at baseline (B = 0.381, *p* = 0.039, 95% CI: [0.020, 0.741]), and GLP1-RA (B = 0.081, *p* = 0.471, 95% CI: [−0.143, 0.305]). Details are provided in Table S6 in the Supplementary Material.

### 3.5. Intravitreal Injections

As described in the Methods section, each classification of IVI response was based on several clinical categories, and the various responses in the two study groups are summarized in Table 3. For simplification and due to the relatively small sample size, a binary IVI response by pooling “Good” and “Stable” responses for subsequent analyses was generated (Table 4).

When comparing the change in CST based on IVI response, no difference was found between Good/Stable responders and Poor responders (−30.18 ± 14.362 vs. 3.55 ± 17.513 microns, respectively; *p* = 0.267, Student’s *t*-test). A comparison of IVI responses for all other measured OCT parameters was also performed, but no baseline OCT parameters were found to be associated with the response to IVI (Table 4). After follow-up, only SRF was significantly associated with poor IVI response, as expected.

IVI response did not differ significantly between patients who had laser therapy, vitrectomy, or a new incidence during follow-up (Supplementary Material Table S7). All baseline variables were also compared, as summarized in Supplementary Material Table S4, revealing that aspirin use (17/22 of poor responders vs. 22/44 of good/stable responders; *p* = 0.034, Pearson’s chi-square) was associated with a good/stable response. At baseline, having relatively good VA (0.35 vs. 0.69 LogMAR for good/stable and poor responders, respectively; *p* = 0.010, Student’s *t*-test), lower DR stage (3.1 vs. 2.8 for good/stable and poor responders, respectively; *p* = 0.051, Fisher-Freeman-Halton test), and HRF (as described above) were all associated with a poor IVI response. Two baseline ocular conditions—retinal detachment (present in 8/44 vs. 0/22 of good/stable and poor responders, respectively; *p* = 0.045, Fisher’s exact test) and vitreous hemorrhage at baseline (present in 19/44 vs. 3/22 of good/stable and poor responders, respectively; *p* = 0.016, Fisher’s exact test)—were associated with a good IVI response. However, a multivariable quantitative model with stepwise selection included only vitreous hemorrhage (Exp(B) = 0.208, *p* = 0.023, 95% CI: [0.054, 0.806]), with none of these conditions or other baseline characteristics being significant afterward. This constructed model was a naïve model, with 66.7% accuracy, 100% sensitivity, and 0% specificity. Thus, because vitreous hemorrhage did not result in a good fit to the model, the same process was repeated after excluding this variable, this time yielding an improved model with 78.8% accuracy, 90.9% sensitivity, and 54.5% specificity for a good/stable IVI response. This model included the variables aspirin (Exp(B) = 5.030, *p* = 0.021, 95% CI: [1.272, 19.886]), VA at baseline (Exp(B) = 0.255, *p* = 0.048, 95% CI: [0.066, 0.989]), HRF (Exp(B) = 2.919, *p* = 0.136, 95% CI: [0.713, 11.9444]), and GLP1-RA (Exp(B) = 0.317, *p* = 0.082, 95% CI: [0.087, 1.155]).

Based on the between-group comparisons shown in Table 1, vitreous hemorrhage and HRF are expected to have a confounding association between IVI response and GLP1-RA use. IVI response rates between study groups seem to differ when stratified by HRF presence. With HRF absent at baseline, GLP1-RA treated patients had a significantly higher percentage of good responders (26/31 vs. 11/20, OR: 0.235, *p* = 0.026, 95% C.I: [0.064, 0.863], GLP1-RA and control patients respectively). Although among those with HRF at baseline, GLP1-RA patients had a much lower good responders rate compared to unchanged rates in the control group (1/4 vs. 6/11, OR: 3.600, *p* = 0.999, 95% CI: [0.280, 46.359], GLP1-RA and control patients respectively). Meanwhile, stratification of IVI response by vitreous hemorrhage shows that the link described above is extensively related to its presence at baseline (14/16 vs. 5/6, OR: 0.714, *p* = 0.999, 95% CI: [0.053, 9.700], GLP1-RA and control patients, respectively), with lower response rates in its absence (13/19 vs. 12/25, OR: 0.426, *p* = 0.176, 95% C.I: [0.123, 1.480], GLP1-RA and control patients, respectively). To further assess their potential effects as confounders, we also generated a simultaneous entry logistic regression model consisting of GLP1-RA and only these two factors. When taking only vitreous hemorrhage and HRF into account as variables, the adjusted OR for GLP1-RA was no longer significant (adjusted OR: 1.76, 95% CI: [0.553, 5.650], *p* = 0.229, logistic regression). In addition, although vitreous hemorrhage (OR: 3.802, 95% CI: [0.936, 15.385], *p* = 0.062) and HRF (OR: 0.453, 95% CI: [0.125, 1.645], *p* = 0.116) had larger effect sizes, they were not significant.

### 3.6. OCT Parameters

Central subfield thickness (CST), as well as the presence of microaneurysms, exudates, intra- and subretinal fluid (IRF and SRF), and HRF, were documented based on OCT examinations performed both at baseline and at the last follow-up (Table 5). None of the measured variables differed significantly between the study groups during follow-up, although a weak association between the GLP1-RA group and IRF resolution was observed, with IRF resolution observed in 7/22 and 2/25 patients in the GLP1-RA and control groups, respectively (*p* = 0.106, Fisher’s exact test).

At baseline, CST differed significantly between the GLP1-RA and control groups (305.54 ± 12.89 vs. 362.55 ± 20.67 microns, respectively; *p* = 0.020, Student’s *t*-test). However, the change in CST during follow-up was similar between the GLP1-RA and control groups (with a mean change of −13.49 vs. −30.13 microns, respectively; *p* = 0.471, Student’s *t*-test). Moreover, high CST at baseline was associated with a reduction in CST during follow-up (Pearson’s correlation coefficient: −0.674, *p* < 0.001). Other important baseline variables related to the change in CST during follow-up were the use of statins vs. not using statins (−0.6 ± 68.5 vs. −91.8 ± 122.9 microns, respectively; *p* = 0.014, Student’s *t*-test), the presence or absence of microaneurysms (−16.9 ± 89.2 vs. −161.0 ± 35.4 microns, respectively; *p* = 0.027, Student’s *t*-test), and the presence or absence of SRF at baseline (−152.7 ± 161.6 vs. −15.0 ± 83.9 microns, respectively; *p* = 0.010, Student’s *t*-test).

A quantitative dependent linear regression model with stepwise selection was performed to further analyze the effect of variables on the change in CST. The variables baseline CST (B = −0.5, *p* < 0.001, 95% CI: [−0.7, −0.4]), the use of statins (B = 74.3, *p* < 0.001, 95% CI: [40.6, 108.0]), dialysis (B = −75.7, *p* = 0.004, 95% CI: [−125.6, −25.8]), and the presence of an epiretinal membrane (B = 49.0, *p* = 0.011, 95% CI: [11.7, 86.2]) were statistically significant. In contrast, GLP1-RA treatment was not statistically significant (partial correlation: −0.169, *p* = 0.189). The model performance had an R^2^ value of 0.645 and a standard error of 56.2.

## 4. Discussion

In this study, DR progression among patients with non-proliferative DR at baseline was slightly—but not significantly—associated with GLP1-Ra treatment. Although changes in VA and CST were similar between the study groups, our analysis demonstrated an association between GLP1-RA treatment and a good or stable response to IVI therapy. However, further examination revealed that the presence of vitreous hemorrhage and hyperreflective retinal foci confounded this effect. Multivariable models for VA, CST, and IVI response were generated, but only the model for IVI response was informative.

Previous studies were either relatively small and retrospective or focused primarily on major cardiovascular events without conducting a comprehensive ocular evaluation [9,12,13,15,16]. The first large randomized controlled trial (RCT) to comprehensively examine ocular outcomes in patients with DR receiving GLP1-RA is the FOCUS trial [17], the results of which are expected to be available in 2027. The observed ocular effects of GLP1-RA in previously published studies are varied and inconclusive [9], and the response to IVIs and OCT parameters has not previously been considered.

**DR progression:** The observed rate of DR progression in the GLP1-RA group (3/19 patients, 15%) is higher than that in several previous studies [9,10,12,13,14]. One reason for this difference may be baseline differences between patient populations. While the present study included a relatively small cohort of patients with baseline DR who underwent OCT scans and/or color fundus exams, other studies focused on patients with T2DM and their cardiovascular complications in general, resulting in only a limited focus on DR and vastly different patient profiles, with lower rates of advanced DR, OCT scans, and color fundoscopies at baseline [9,11,12,13,14,15,16]. Studies published regarding GLP1-RA and DR refer primarily to DR progression in populations of patients with T2DM before a diagnosis of DR or include a small percentage of patients with DR at baseline. Bethel et al. performed a meta-regression analysis and found an overall OR of 1.10 (95% CI: [0.92, 1.30], *p* = 0.290) and high heterogeneity between studies (*I*^2^ = 52.22%) [9]. The reported rate of DR progression ranged from 3.0% (50/1648) in the GLP1-RA group and 1.9% (92/4672) in the control group in the SUSTAIN-6 trial [13], implying that GLP1-RA tends to worsen DR, to 1.6% (78/4731) in the GLP1-RA group and 1.9% (89/4732) in the control group in the HARMONY trial [10], suggesting the opposite trend. In another recently published retrospective cohort study by Joo et al. on 692 GLP1-RA patients with a year of follow-up, the DR clinical worsening rate was 2.3%. Propensity score-based comparison to SGLT2i users who served as the control (OR = 0.33, 95% C.I [0.110, 1.03]), demonstrated no relative association of GLP1-RA with clinical DR worsening [12]. Patient distribution in this study had less advanced DR, and OCT imaging was not an inclusion requirement compared with our study [12].

It is important to note that the estimated annual risk of DR progression varied significantly based on DR severity, as patients with moderate DR have a 12–27% risk of progression, and the risk is as high as 52% for patients with severe NPDR [2]. Those estimations are consistent with the rates of DR progression measured in our study.

**Visual acuity:** The change in VA during follow-up was not significant between the GLP1-RA and control groups (−0.14 ± 0.13 vs. −0.06 ± 0.09 LogMAR, respectively; *p* = 0.664). This is not surprising, however, as ocular conditions and treatments such as laser therapy may have a much higher effect, and in a relatively small cohort such as this one, the statistical power appears to be too low to overcome these higher effects. It is also important to note that a LogMAR change of <0.2 can be measured due to technical variations during measurement and may not necessarily be clinically relevant [23].

**IVI response:** There are currently no published clinical data regarding the effects of GLP1-RA medications on the IVI treatment course. Here, we found a slightly better response to IVIs in the GLP1-RA group than in the control group (27/35 vs. 17/31, respectively; *p* = 0.055), with an unadjusted OR of 2.779 (95% CI: [0.963, 8.020]; *p* = 0.058). However, this difference is likely due primarily to baseline differences in the prevalence of vitreous hemorrhage between the GLP1-RA and control groups (16/35 vs. 6/31, respectively; *p* = 0.023). After adjusting for this variable, the adjusted OR dropped to 1.767 (95% CI: [0.553, 5.650]) and was no longer statistically significant (*p* = 0.336).

At baseline, vitreous hemorrhage was present in 43.2% (19/44) of good/stable IVI responders compared to only 13.6% (3/22) of poor responders (*p* = 0.016). However, this association between vitreous hemorrhage at baseline and a good/stable response to IVIs is not surprising, as IVI is one of the treatment options commonly available to patients with this condition [24].

Hyperreflective retinal foci (HRF) are defined as hyperreflective dots or roundish lesions within the retinal layers visible on OCT and are believed to represent extravasated protein, lipid deposits, and/or macrophages. HRF can be considered a nonspecific biomarker of progression for several retinal diseases [25]. Interestingly, previous studies investigating the value of HRF in predicting visual outcomes in diabetic macular edema treated with anti-VEGF agents yielded conflicting conclusions [25,26].

Herein, we found that HRF was weakly associated with a poor response to IVI; specifically, HRF was present in 36.4% (8/22) of poor responders compared to only 15.9% (7/44) of good/stable responders (*p* = 0.062). However, the presence of HRF also differed significantly between the GLP1-RA and control groups at baseline (4/35 vs. 11/31, respectively; *p* = 0.02).

**OCT findings:** Regarding the effect of GLP1-RA on DME occurrence, there is currently only mechanistic evidence for the neuroprotective [6,7] and anti-inflammatory [8] blood-retinal barrier processes. However, the observed changes in CST were minor in both groups and did not differ significantly between the GLP1-RA and control groups (−13.5 ± 13.5 vs. −30.1 ± 18.5 microns, respectively; *p* = 0.464).

Finally, recent data suggests that GLP1-RAs have been associated with a higher incidence of non-arteritic, non-ischemic optic neuropathy [27]. We did not have any cases of NAION in our cohort.

## 5. Limitations

Although this is the first study to include only patients with medical records and OCT exam data, and the first to measure the association between GLP1-RA treatment course and IVI response, this study has several limitations that warrant discussion.

**General study design:** First, the retrospective nature of the study is a limitation. Only 66 patients (35 in the GLP1-RA group and 31 in the control group) were included in the final analysis, which limits the study’s statistical power. The follow-up duration of 1 year is relatively short, and only three incidences of DR progression occurred, which is too few for robust statistical analysis. Additionally, the GLP1-RA group included thirty-three patients on liraglutide and two on Semaglutide, so only these GLP1-RA medications were studied. Therefore, the findings are preliminary and larger, more robust studies with longer follow-ups and broader coverage of GLP1-RA analogs are needed to provide further insights.

**Information bias:** After manually reviewing patient records, up to four patients were excluded due to incorrect information discovered during this stage. Reasons for excluding these patients were previous GLP1-RA documentation that was not evident during the technical search phase and a diagnosis of type 1 diabetes mellitus (T1DM) instead of T2DM. This finding may raise two additional potential biases: (1) several patients who received GLP1-RA medication may have been missed during the technical search phase, and (2) the medical records of the included patients may have contained inaccuracies that went undetected.

**Selection bias:** Including only patients who underwent at least two OCT exams may have resulted in the selection of patients who tend to be sicker, have a higher rate of comorbidity, and/or have a higher rate of advanced ocular disease at baseline compared to the general population.

### Study Strengths

This is the first study to combine medical records with ophthalmological examinations—including OCT exams—for all patients. Furthermore, the OCT scans were interpreted in a blinded, masked fashion. It is also the first study to examine the effect of GLP1-RA on the response to IVIs. Despite limitations in power and design, this study highlights the importance of performing a comprehensive analysis of patients with DR. The integration of OCT data, information regarding invasive ocular treatments, and detailed descriptions of IVI treatments in this study provide the first evidence of both a possible association between GLP1-RA treatment and a good/stable response to IVIs, as well as the possible confounding role of HRF, an OCT parameter that has not been considered previously in this context.

## Figures and Tables

**Table 1:** Baseline variables in the two study groups.

Category	Variable		GLP1-RA (N = 35)	Control (N = 31)	*p*-Value
Epidemiological Parameters	Age (years)		63.3 ± 1.7	66.0 ± 1.7	0.266 ^a^
	Sex	F	16 (45.7)	14 (45.2)	0.964 ^b^
		M	19 (54.3)	17 (54.8)	
	Follow-up duration (days)		599.0 ± 75.9	404.8 ± 21.1	**0.017 ^c^**
T2DM Characteristics	BMI (kg/m^2^)		28.8 ± 0.4	28.0 ± 0.6	0.250 ^a^
	HbA1c (%), 1st		7.9 ± 0.2	7.8 ± 0.4	0.770 ^a^
	T2DM duration (years)		13.9 ± 1.6	12.4 ± 1.1	0.459 ^a^
DR Characteristics	DR duration (years)		2.7 ± 0.6	2.8 ± 0.5	0.303 ^c^
	DR Disease Severity Scale	Mild NPDR	2 (5.7)	0	0.469 ^d^
		Moderate NPDR	11 (31.4)	12 (38.7)	
		Severe NPDR	6 (17.1)	8 (25.8)	
		PDR	16 (45.7)	11 (35.8)	
	Visual acuity (logMAR)		0.66 ± 0.12	0.49 ± 0.10	0.266 ^a^
	Laser therapy		19 (54.3)	14 (45.2)	0.459 ^b^
	Anti-VEGF IVIs		21 (60.0)	28 (90.3)	**0.050 ^b^**
	Vitrectomy		7 (20.0)	2 (6.5)	0.156 ^e^
Other Risk Factors	Tobacco use		13 (37.1)	6 (19.4)	0.111 ^b^
	Hypertension		29 (82.9)	24 (77.4)	0.579 ^b^
	Hyperlipidemia		26 (74.3)	21 (67.7)	0.558 ^b^
Diabetes-related Complications	CVD		24 (68.6)	18 (58.1)	0.376 ^b^
	PVD		13 (37.1)	10 (32.3)	0.993 ^b^
	Nephropathy		21 (60.0)	16 (45.7)	0.635 ^b^
	Neuropathy		16 (45.7)	12 (38.7)	0.566 ^b^
Medications	Insulin		29 (82.9)	20 (64.5)	0.089 ^b^
	SGLT2 inhibitor		15 (42.9)	8 (25.8)	0.147 ^b^
	Aspirin		23 (65.7)	16 (51.6)	0.245 ^b^
Ocular Conditions	Retinal detachment		3 (8.6)	5 (16.1)	0.459 ^e^
	Vitreous hemorrhage		16 (45.7)	6 (19.4)	**0.023 ^b^**
OCT Findings	Microaneurysms		35 (100.0)	31 (93.5)	0.217 ^e^
	Exudates		3 (8.6)	4 (12.9)	0.698 ^e^
	CST (microns)		305.5 ± 12.9	362.6 ± 20.7	**0.020 ^a^**
	IRF		22 (62.9)	25 (80.6)	0.111 ^b^
	SRF		1 (2.9)	2 (6.5)	0.597 ^e^
	HRF		4 (11.4)	11 (35.5)	**0.020 ^b^**

**Statistical tests used**: ^a^, Student’s *t*-test; ^b^, Pearson’s chi-square; ^c^, Mann–Whitney *U* test; ^d^, Fisher–Freeman–Halton exact test; ^e^, Fisher’s exact test. **Abbreviations**: GLP1-RA, glucagon-like peptide-1 receptor agonist; BMI, body mass index; T2DM, type 2 diabetes mellitus; DR, diabetic retinopathy; NPDR, non-proliferative DR; PDR, proliferative DR; HbA1c, hemoglobin A1c; IVIs, intravitreal injections; CVD, cardiovascular disease; logMAR, logarithm of the minimum angle of resolution; PVD, peripheral vascular disease; CST, central subfield thickness; IRF, intraretinal fluid; SRF, subretinal fluid; HRF, hyperreflective retinal foci; OCT, optical coherence tomography; SGLT2, sodium-glucose transport protein 2. Data are presented as N (%) or mean ± SD. Diabetic Retinopathy Disease Severity Scale: 1: Mild non-proliferative diabetic retinopathy (NPDR); 2: Moderate NPDR; 3: Severe NPDR; 4: Proliferative diabetic retinopathy.

**Table 2:** Outcomes in each study group.

Measured Outcome		GLP1-RA (N = 35)	Control (N = 31)	*p*-Value
Primary Outcome				
DR progression	Progression	3 (15.8)	0	0.106 ^a^
	No progression	16 (84.2)	20 (100)	
IVIs response	Good/Stable *	27 (77.1)	17 (54.8)	0.055 ^b^
	Poor	8 (22.9)	14 (45.2)	
Change in VA (logMAR)		−0.14 ± 0.13	−0.06 ± 0.09	0.664 ^c^
Secondary Outcome				
Change in CST (microns)		−13.5 ± 13.5	−30.1 ± 18.5	0.464 ^c^
Ocular Surgery **				
Vitrectomy during follow-up		2 (7.1)	3 (10.3)	0.999 ^a^
Laser therapy during follow-up		6 (37.5)	3 (17.6)	0.227 ^a^

**Statistical tests:** ^a^, Fisher’s exact Test; ^b^, Pearson’s chi-square; ^c^, Student’s *t*-test. **Abbreviations**: GLP1-RA, glucagon-like peptide-1 receptor agonist; IVI, intravitreal injection; VA, visual acuity; logMAR, logarithm of the minimum angle of resolution; CST, central subretinal thickness. Data are presented as N (%) or mean ± SD. * In this table, “Good” and “Stable” responders were pooled into one category. ** The percentage of ocular surgeries was calculated relative to the number of patients who had not undergone this surgery at the start of follow-up. Data are presented as N (%) or mean ± SD.

**Table 3:** Summary of response to anti-VEGF IVIs.

IVI-Related Clinical Category	GLP1-RA (N = 35)	Control (N = 31)
**Good responders (total)**	**14 (40.0)**	**6 (19.4)**
Tx remission	8	5
Longer interval between IVIs	6	1
**Stable responders (total)**	**13 (37.1)**	**11 (35.5)**
Same Tx	4	7
Not treated	9	4
**Poor responders (total)**	**8 (22.9)**	**14 (45.1)**
Shorter interval between IVIs	1	3
Changed to an advanced Tx	2	8
Tx started during follow-up	5	3

**Abbreviations**: GLP1-RA, glucagon-like peptide-1 receptor agonist; IVI, intravitreal injection; Tx, treatment. Data are presented as N (%).

**Table 4:** Summary of OCT parameters with a Good/Stable or Poor IVI response at baseline and the end of follow-up.

OCT Parameter	Good/Stable IVI Response (N = 44)		Poor IVI Response (N = 22)		*p*-Value	
	Baseline	Follow-Up	Baseline	Follow-Up	Baseline	Follow-Up
Microaneurysms	43 (97.7)	42 (95.4)	21 (95.5)	21 (95.5)	0.999 ^a^	0.999 ^a^
Exudates	4 (9.1)	8 (18.2)	3 (13.6)	4 (18.2)	0.678 ^b^	0.999 ^b^
IRF	31 (70.5)	26 (59.1)	16 (72.7)	17 (77.3)	0.848 ^a^	0.144 ^a^
SRF	1 (2.3)	0	2 (9.1)	3 (13.6)	0.256 ^b^	0.034 ^b^
HRF	7 (15.9)	8 (18.2)	8 (36.2)	5 (22.7)	0.062 ^a^	0.746 ^a^

**Abbreviations**: OCT, optical coherence tomography; IVI, intravitreal injection; IRF, intraretinal fluid; SRF, subretinal fluid; HRF, hyperreflective retinal foci. **Statistical tests used:** ^a^, chi-square Test; ^b^, Fisher’s exact test. Data are presented as N (%).

**Table 5:** Summary of OCT categorical parameters at baseline and the end of follow-up in both study groups.

OCT Parameter	GLP1-RA (n = 35)			Control (n = 31)			*p*-Value
	Baseline	Resolved *	New Onset **	Baseline	Resolved *	New Onset **	
Microaneurysms	35 (100.0)	1 (2.9)	0	31 (93.5)	0	0	0.999
Exudates	3 (8.6)	1 (33.3)	3 (9.4)	4 (12.9)	0	3 (11.1)	0.999
IRF	22 (62.9)	7 (31.8)	4 (30.8)	25 (80.6)	2 (8.0)	1 (16.7)	0.120
SRF	1 (2.9)	1 (100)	0	2 (6.5)	0	1 (3.2)	0.723
HRF	4 (11.4)	3 (75)	1 (3.2)	11 (35.5)	1 (9.1)	1 (3.3)	0.806

**Abbreviations**: OCT, optical coherence tomography; IRF, intraretinal fluid; SRF, subretinal fluid; HRF, hyperreflective retinal foci. * The percentage is based on the number of patients with this OCT parameter at baseline. ** The percentage is based on the number of patients who did not present with this OCT parameter at baseline. **Statistical test used:** Fisher-Freeman-Halton exact test. Data are presented as N (%).

## Data Availability

The dataset presented in this article is not readily available because the data is part of an ongoing study. Requests to access the datasets should be directed to Michaeli, T, via mail tomer.michaeli1@mail.huji.ac.il and with Hadassah Medical Center institutional consent.

## Supplementary Materials

The following supporting information can be downloaded at: https://www.mdpi.com/article/10.3390/jcm13206269/s1, Table S1: Full comparison between study groups at baseline; Table S2: Full comparison of measured outcomes between study groups; Table S3: Incidence of ocular surgeries per DR Progression and IVI Response; Table S4: IVI response (Good/Stable or Poor) per baseline characteristic; Table S5: Change in CST per baseline characteristics; Table S6: Change in visual acuity per baseline variable; Table S7: Change in visual acuity and change in CST per incidence of ocular surgeries.
