# Supplementary material for: The Effect of Glucagon-like-Peptide-1 Receptor Agonists on Diabetic Retinopathy Progression, Central Subfield Thickness, and Response to Intravitreal Injections
Source: J Clin Med. 2024 Oct 21;13(20):6269. doi: 10.3390/jcm13206269 (PMC11508636; doi:10.3390/jcm13206269)
Supplement: Supplementary file 1 [file jcm-13-06269-s001.zip › jcm-3212149-supplementary.pdf]

Supplementary Materials

Statistical tests used in all tables: a, Student's *t*-test; b, Pearson's chi-square test; c, Fisher-Freeman-Halton test; d, Mann-Whitney *U* test; e, Fisher's exact test; f, Pearson's correlation coefficient; g, Spearman's correlation coefficient; h, Kruskal-Wallis test.

In all tables, data are presented as N (%) or the mean ± SD.

Table S1: Full comparison between study groups at baseline

Category	Variable	GLP1-RA (N=35)	Control (N=31)	*p*-value
Epidemiological Parameters	Age	63.3±1.7	66.0±1.7	0.266[a]
	Sex F	16 (45.7)	14 (45.2)	0.964[b]
	M	19 (54.3)	17 (54.8)	
	Origin Israel	25	22	0.990[c]
	Middle East	7	6	
	Europe and the Americas	3	3	
	Follow-up (days)	599.0±75.9	404.8±21.1	0.017[d]
T2DM Characteristics	BMI (kg/m^2)	28.8±0.4	28.0±0.6	0.250[a]
	HbA1c (%), 1st	7.9±0.2	7.8±0.4	0.770[a]
	T2DM duration (years)	13.9±1.6	12.4±1.1	0.459[a]
DR Characteristics	DR duration	2.7±0.6	2.8±0.5	0.303[d]
	DR severity, Mild NPDR	2 (5.7)	0	0.469[c]
	Moderate NPDR	11 (31.4)	12 (38.7)	
	Severe NPDR	6 (17.1)	8 (25.8)	
	PDR	16 (45.7)	11 (35.8)	
	Visual acuity (LogMAR)	0.66±0.12	0.49±0.10	0.266[a]
	Laser therapy	19 (54.3)	14 (45.2)	0.459[b]
	IVIs	21 (60.0)	28 (90.3)	0.050[b]
	Vitrectomy	7 (20.0)	2 (6.5)	0.156[e]
Other Risk Factors	Tobacco use	13 (37.1)	6 (19.4)	0.111[b]
	Hypertension	29 (82.9)	24 (77.4)	0.579[b]
	Hyperlipidemia	26 (74.3)	21 (67.7)	0.558[b]
Diabetes Complications	IHD	20 (57.1)	12 (38.7)	0.135[b]
	CHF	9 (25.7)	8 (25.8)	0.993[b]
	PVD	13 (37.1)	10 (32.3)	0.678[b]
	Diabetic ulcer	10 (28.6)	5 (16.1)	0.229[e]
	Amputation	2 (5.7)	1 (3.2)	0.990[e]
	Diabetic nephropathy	16 (45.7)	16 (51.6)	0.635[b]
	CKD	15 (42.9)	8 (25.8)	0.147[b]
	Dialysis	2 (5.7)	4 (12.9)	0.408[e]
	Diabetic neuropathy	16 (45.7)	6 (19.4)	0.566[b]
	Neurovascular disease	8 (22.9)	6 (19.4)	0.728[b]
Medications	Insulin	29 (82.9)	20 (64.5)	0.089[b]
	glimepiride	5 (14.3)	9 (29.0)	0.147[b]
	Alpha-glucosidase	1 (2.9)	0	0.245[e]
	Thiazolidinedione	1 (2.9)	0	0.990[e]
	SGLT2 inhibitor	15 (42.9)	8 (25.8)	0.147[b]
	Aspirin	23 (65.7)	16 (51.6)	0.245[b]
	Ezetimibe	3 (8.6)	3 (9.7)	0.999[e]
	Statins	28 (80.0)	23 (74.2)	0.574[b]

	Anticoagulation	9 (25.7)	4 (12.9)	0.192[e]
	Beta-blockers	20 (57.1)	16 (51.6)	0.652[b]
	Diuretics	16 (45.7)	11 (35.5)	0.399[b]
Ocular Conditions	Retinal detachment	3 (8.6)	5 (16.1)	0.459[e]
	Vitreous hemorrhage	16 (45.7)	6 (19.4)	0.023[b]
	Glaucoma	10 (28.6)	5 (16.1)	0.229[b]
	Pseudophakia	18 (51.4)	19 (61.3)	0.420[b]
	Retinal atrophy	1 (2.9)	1 (3.2)	0.999[e]
	Epiretinal membrane	4 (11.4)	7 (22.6)	0.225[b]
OCT Findings	Microaneurysms	35 (100.0)	31 (93.5)	0.217[e]
	Exudates	3 (8.6)	4 (12.9)	0.698[e]
	CST (microns)	305.5±12.9	362.6±20.7	0.020[a]
	IRF	22 (62.9)	25 (80.6)	0.111[b]
	SRF	1 (2.9)	2 (6.5)	0.597[e]
	HRF	4 (11.4)	11 (35.5)	0.020[b]

Abbreviations: GLP1-RA, glucagon-like peptide-1 receptor agonist; T2DM, type 2 diabetes mellitus; DR, diabetic retinopathy; NPDR, non-proliferative diabetic retinopathy; PDR, proliferative diabetic retinopathy; BMI, body mass index; HbA1c, hemoglobin A1c; LogMAR, Logarithm of the minimum angle of resolution; IVIs, intravitreal injections; IHD, ischemic heart disease; CHF, congestive heart failure; PVD, peripheral vascular disease; CKD, chronic kidney disease; OCT, optical coherence tomography; IRF, intraretinal fluid; SRF, subretinal fluid; HRF, hyperreflective retinal foci.

Table S2: Full comparison of measured outcomes between study groups

Measured Outcome		GLP1-RA (N=35)	Control (N=31)	*p*-value
**Primary Outcomes**				
DR progression	Progression	3 (15.8)	0	0.106[e]
	No progression	16 (84.2)	20 (100)	
IVI response	Good/stable	27 (77.1)	17 (54.8)	0.055[b]
	Poor	8 (22.9)	14 (45.2)	
VA change (LogMAR)		-0.14±0.13	-0.06±0.09	0.664[a]
**Secondary Outcomes**				
CST change (microns)		-13.5±13.5	-30.1±18.5	0.464[a]
**Ocular Surgeries***				
**Vitrectomy**				
At baseline		7 (20.0)	2 (6.5)	0.156[e]
During follow-up (all):		4 (11.4)	3 (9.7)	0.999[e]
First time		2 (13.3)	3 (10.3)	0.999[e]
Recurrence		2 (28.6)	0	0.999[e]
**Laser therapy**				
At baseline:		19 (54.3)	14 (45.2)	0.459[b]
During follow-up (all):		15 (42.9)	9 (29.0)	0.244[b]
First time		6 (37.5)	3 (17.6)	0.201[e]
Recurrence		9 (47.4)	6 (42.9)	0.227[b]

Abbreviations: GLP1-RA, glucagon-like peptide-1 receptor agonist; DR, diabetic retinopathy; logMAR, logarithm of the minimum angle of resolution; IVI, intravitreal injection; CST, central subfield thickness.

Table S3: Incidence of ocular surgeries per DR Progression and IVI Response

	DR Progression			IVI Response		
	**Yes (N=3)**	**No (N=36)**	**p-value**	**Good/Stable (N=44)**	**Poor (N=22)**	***p*-value**
**Vitrectomy**						
At Baseline	1 (33.3)	2 (5.6)	0.219[e]	7 (15.9)	2 (9.1)	0.706[e]
During Follow-up (all):	1 (33.3)	0	0.077[e]	6 (13.6)	1 (4.5)	0.409[e]
First Time	1 (33.3)	0	0.056[e]	4 (11.4)	1 (5.0)	0.647[e]
Recurrence	0	0	-	2 (28.6)	0	0.999[e]
**Laser**						
At Baseline:	1 (33.3)	10 (27.8)	0.642[e]	25 (56.8)	8 (36.4)	0.117[b]
During Follow-up (all):	2 (66.7)	6 (16.7)	0.101[e]	15 (34.1)	9 (4.1)	0.587[b]
First Time	2 (66.7)	3 (11.5)	0.027[e]	5 (26.3)	4 (28.6)	0.999[e]
Recurrence	0	3 (30.0)	0.999[e]	10 (40.0)	5 (62.5)	0.418[e]

Abbreviations: DR, diabetic retinopathy; IVIs, intravitreal injections;

Table S4: IVI response (Good/Stable or Poor) per baseline characteristic

Category	Variable	Good/Stable IVI Response (N=44)	Poor IVI Response (N=22)	*p*-value
Epidemiological Parameters	Age	64.0±1.5	65.6±2.1	0.539[a]
	Sex F	21 (47.7)	9 (40.9)	0.600[b]
	M	23 (52.3)	13 (59.1)	
	Origin Israel	33 (75.0)	14 (63.6)	0.580[c]
	Middle East	8 (18.2)	5 (22.7)	
	Europe and Americas	3 (6.8)	3 (13.6)	
	Follow-up (days)	513.8±55.5	495.7±66.9	0.759[d]
T2DM Characteristics	BMI (kg/m^2)	28.5±0.5	28.3±0.6	0.780[a]
	HbA1c (%), 1st	8.0±0.2	7.6±0.4	0.356[a]
	T2DM duration	13.4±1.3	12.9±1.5	0.812[a]
DR Characteristics	DR duration	2.80±0.49	2.77±0.49	0.557[a]
	DR severity, 1	2 (4.5)	0	0.051[c]
	2	14 (31.8)	9 (40.9)	
	3	6 (13.6)	8 (36.4)	
	4	22 (50.0)	5 (22.7)	
	Visual acuity(logMAR)	0.70±0.11	0.35±0.08	0.010[a]
	Laser therapy			
	IVIs	25 (56.8)	8 (36.4)	0.117[b]
	Vitrectomy	33 (75.0)	16 (72.7)	0.842[b]
		7 (15.9)	2 (9.1)	0.706[e]
Diabetes Complications	IHD	24 (54.5)	8 (36.4)	0.164[b]
	CHF	12 (27.3)	5 (22.7)	0.691[b]
	PVD	14 (31.8)	9 (40.9)	0.465[b]
	Diabetic ulcer	10 (22.7)	5 (22.7)	0.999[e]
	Amputation	2 (4.5)	1 (4.5)	0.999[e]
	Diabetic nephropathy	23 (52.3)	9 (40.9)	0.384[b]
	CKD	18 (40.9)	5 (22.7)	0.144[b]
	Dialysis	5 (11.4)	1 (4.5)	0.655[e]
	Diabetic neuropathy	18 (40.9)	10 (45.5)	0.725[b]
	Neurovascular disease	9 (20.5)	5 (22.7)	0.990[b]
Medications	Insulin	32 (72.7)	17 (77.3)	0.089[b]
	glimepiride	9 (20.5)	5 (22.7)	0.147[b]
	Alpha-glucosidase	1 (2.3)	0	0.245[e]
	Thiazolidinedione	1 (2.3)	0	0.990[e]
	SGLT2 inhibitor	14 (31.8)	9 (40.9)	0.147[b]
	Aspirin	22 (50.0)	17 (77.3)	0.245[b]
	Ezetimibe	3 (6.8)	3 (13.6)	0.999[e]
	Statins	32 (72.7)	19 (86.4)	0.574[b]
	Anticoagulation	8 (18.2)	5 (22.7)	0.192[e]
	Beta-blockers	25 (56.8)	11 (50.0)	0.652[b]
	Diuretics	18 (40.9)	9 (40.9)	0.399[b]
Ocular Conditions	Retinal detachment	8 (18.2)	0	0.045[e]
	Vitreous hemorrhage	19 (43.2)	3 (13.6)	0.016[b]
	Glaucoma	11 (25.0)	4 (18.2)	0.533[b]
	Pseudophakia	24 (54.5)	13 (59.1)	0.726[b]

	Retinal atrophy	0	2 (9.1)	0.108[e]
	Epiretinal membrane	7 (15.9)	4 (18.2)	0.990[b]
OCT Findings	Microaneurysms	43 (97.7)	21 (95.5)	0.999[e]
	Exudates	4 (9.1)	3 (13.6)	0.678[e]
	CST (microns)	-30.2±14.6	3.5±17.4	0.267[a]
	IRF	31 (70.5)	16 (72.7)	0.848[b]
	SRF	1 (2.3)	2 (9.1)	0.256[e]
	HRF	7 (15.9)	8 (36.4)	0.062[b]

Abbreviations: GLP1-RA, glucagon-like peptide-1 receptor agonist; T2DM, type 2 diabetes mellitus; DR, diabetic retinopathy; BMI, body mass index; HbA1c, hemoglobin A1c; LogMAR, Logarithm of the minimum angle of resolution; IVIs, intravitreal injections; IHD, ischemic heart disease; CHF, congestive heart failure; PVD, peripheral vascular disease; CKD, chronic kidney disease; OCT, optical coherence tomography; IRF, intraretinal fluid; SRF, subretinal fluid; HRF, hyperreflective retinal foci.

Table S5: Change in CST per baseline characteristics

Category	Variable		N (%)	CST Change (microns)	p-value
Epidemiological Parameters	Age		66 (100)	0.006	0.964[f]
	Sex	F	30 (45.5)	-3.8±68.9	0.157[a]
		M	36 (54.5)	-35.9±105.3	
	Origin	Israel	47 (71.2)	-26.6±101.1	0.688[h]
		Middle East	13 (19.7)	-21.4±63.1	
		Europe and America	6 (9.1)	20.3±50.8	
	Follow-up (days)		66 (100)	-0.045	0.721[f]
T2DM Characteristics	BMI (kg/m^2)		66 (100)	0.120	0.337[f]
	HbA1c (%), 1st		66 (100)	0.136	0.276[f]
	T2DM duration (days)		66 (100)	0.167	0.181[f]
DR Characteristics	DR duration		66 (100)	0.045	0.721[f]
	DR severity,	1	2 (3.0)	3.0±5.7	0.711[h]
		2	23 (34.8)	-29.5±84.0	
		3	14 (21.2)	-2.6±110.5	
		4	27 (40.9)	-25.9±92.1	
	Visual acuity		66 (100)	-0.100	0.423[f]
	Laser Therapy	Yes	33 (50.0)	-24.2±68.3	0.802[a]
		No	33 (50.0)	-18.5±110.8	
	IVIs	Yes	49 (74.2)	-26.7±100.9	0.420[a]
		No	17 (25.8)	-5.8±55.1	
	Vitrectomy	Yes	9 (13.6)	13.1±37.3	0.227[d]
		No	57 (86.4)	-26.7±96.3	
Other Risk Factors	Tobacco use	Yes	19 (28.8)	-9.7±77.6	0.517[a]
		No	47 (71.2)	-26.0±96.7	
	Hypertension	Yes	53 (80.3)	-19.2±98.8	0.702[a]
		No	13 (19.7)	-30.1±53.2	
	Hyperlipidemia	Yes	47 (71.2)	-12.7±77.0	0.233[a]
		No	19 (28.8)	-42.5±119.6	
Diabetes Complications	IHD	Yes	32 (48.5)	-9.7±87.7	0.319[a]
		No	34 (51.5)	-32.3±94.7	
	CHF	Yes	17 (25.8)	-9.2±101.9	0.529[a]
		No	49 (74.2)	-25.5±88.1	
	PVD	Yes	23 (34.8)	-12.9±105.4	0.589[a]
		No	43 (65.2)	-25.8±83.9	
	Diabetic ulcer	Yes	15 (22.7)	-25.1±122.6	0.858[d]
		No	51 (77.3)	-20.0±81.4	
	Amputation	Yes	3 (4.5)	3.0±18.3	0.641[d]
		No	63 (95.5)	-22.5±93.3	
	Nephropathy	Yes	32 (48.5)	-31.3±105.7	0.390[a]
		No	34 (51.5)	-11.9±75.8	
	CKD	Yes	23 (34.8)	-44.3±115.8	0.136[a]
		No	43 (65.2)	-9.0±73.8	
	Dialysis	Yes	6 (9.1)	-127.0±181.0	0.177[d]
		No	60 (90.9)	-10.7±71.9	
	Neuropathy	Yes	28 (42.4)	-19.6±85.4	0.896[a]
		No	38 (57.6)	-22.6±96.6	

	Neurovascular disease	Yes	14 (21.2)	-8.4±49.3	0.554[a]
		No	52 (78.8)	-24.8±99.8	
Medications	Insulin	Yes	49 (74.2)	-26.4±100.6	0.451[a]
		No	17 (25.8)	-6.8±56.9	
	Glimepiride	Yes	14 (21.2)	-10.9±56.8	0.636[a]
		No	52 (78.8)	-24.1±98.9	
	Alpha-glucosidase	Yes	1 (1.5)	7.0	0.758[d]
		No	65 (98.5)	-21.7	
	Thiazolidinedione	Yes	1 (1.5)	16.0	0.684[d]
		No	65 (98.5)	-21.9±91.9	
	SGLT2 inhibitor		23 (34.8)	-8.2±92.7	0.397[a]
	Yes		43 (65.2)	-28.3±90.9	
		No	39 (59.1)	-26.6±112.4	0.524[a]
	Aspirin	Yes	27 (40.9)	-13.6±48.0	
		No	6 (9.1)	18.3±73.0	0.268[d]
	Ezetimibe	Yes	60 (90.9)	-25.3±92.6	
		No	51 (77.3)	-0.6±68.5	0.014[a]
	Statins		15 (22.7)	-91.8±122.9	
	Yes		13 (19.7)	-33.3±100.1	0.601[a]
		No	53 (80.3)	-18.4±89.8	
	Anticoagulation	Yes	36 (54.5)	-26.1±110.2	0.647[a]
		No	30 (45.5)	-15.6±63.0	
	Beta-blockers	Yes	27 (40.9)	3.4±61.9	0.067[a]
		No	39 (59.1)	-38.4±104.5	
	Diuretics	Yes			
		No			
Ocular Conditions	Retinal detachment	Yes	8 (12.1)	-59.8±146.3	0.207[d]
		No	58 (87.9)	-16.0±81.6	
	Vitreous hemorrhage	Yes	22 (33.3)	-5.1±38.6	0.187[a]
		No	44 (66.7)	-29.4±108.1	
	Glaucoma	Yes	15 (22.7)	-12.1±48.1	0.662[a]
		No	51 (77.3)	-24.0±100.8	
	Pseudophakia	Yes	37 (36.1)	-20.8±88.8	0.961[a]
		No	29 (43.9)	-21.9±96.1	
	Retinal atrophy	Yes	2 (3.0)	49.5±0.7	0.269[d]
		No	64 (97.0)	-23.5±91.9	
	Epiretinal membrane	Yes	11 (16.7)	6.9±60.5	0.265[a]
		No	55 (83.3)	-27.0±95.8	
OCT Findings	Microaneurysms	Yes	64 (97.0)	-16.9±89.2	0.027[d]
		No	2 (3.0)	-161.0±35.4	
	Exudates	Yes	7 (10.6)	-12.1±68.1	0.781[d]
		No	59 (89.4)	-22.4±94.1	
	CST		66 (100)	-0.674	<0.001[f]
	IRF	Yes	47 (71.2)	-22.6±86.5	0.854[b]
		No	19 (28.8)	-18.0±104.8	
	SRF	Yes	3 (4.5)	-152.7±161.6	0.010[d]
		No	63 (95.5)	-15.0±83.9	
	HRF	Yes	15 (22.7)	-23.7±78.3	0.910[a]
		No	51 (77.3)	-20.6±95.6	

Abbreviations: GLP1-RA, glucagon-like peptide-1 receptor agonist; T2DM, type 2 diabetes mellitus; DR, diabetic retinopathy; BMI, body mass index; HbA1c, hemoglobin A1c; logMAR, logarithm of the minimum angle of resolution; IVIs, intravitreal injections; IHD, ischemic heart disease; CHF, congestive heart failure; PVD, peripheral vascular disease; CKD, chronic kidney disease; OCT, optical coherence tomography; IRF, intraretinal fluid; SRF, subretinal fluid; HRF, hyperreflective retinal foci.

Table S6: Change in visual acuity per baseline variable

Category	Variable		N (%)	Change in VA (LogMAR)	*p*-value
Epidemiological Parameters	Age		66 (100)	0.037	0.766[f]
	Sex	F	30 (45.5)	-0.21±0.14	0.232[a]
		M	36 (54.5)	-0.01±0.10	
	Origin	Israel	47 (71.2)	-0.05±0.64	0.625[h]
		Middle East	13 (19.7)	-0.14±0.55	
		Europe and the Americas	6 (9.1)	-0.45±1.10	
	Follow-up (days)		66 (100)	-0.141	0.258[g]
T2DM Characteristics	BMI (kg/m^2)		66 (100)	-0.186	0.134[f]
	HbA1c (%), 1st		66 (100)	-0.009	0.940[f]
	T2DM duration (days)		66 (100)	0.158	0.206[f]
DR Characteristics	DR duration		66 (100)	-0.053	0.670[f]
	DR severity, (Baseline)	1	2 (3.0)	0.03±0.10	0.881[h]
		2	23 (34.8)	-0.03±0.48	
		3	14 (21.2)	-0.18±0.85	
		4	27 (40.9)	-0.18±0.67	
	Visual acuity		66 (100)	-0.721	<0.001[f]
	Laser therapy	Yes	33 (50.0)	-0.25±0.13	0.078[a]
		No	33 (50.0)	0.04±0.09	
	IVIs	Yes	49 (74.2)	-0.11±0.11	0.908[a]
		No	17 (25.8)	-0.09±0.10	
	Vitrectomy	Yes	9 (13.6)	-0.31±0.34	0.511[d]
		No	57 (86.4)	-0.07±0.08	
Other Risk Factors	Tobacco use	Yes	19 (28.8)	-0.12±0.19	0.862[a]
		No	47 (71.2)	-0.09±0.09	
	Hypertension	Yes	53 (80.3)	-0.10±0.10	0.961[a]
		No	13 (19.7)	-0.11±0.10	
	Hyperlipidemia	Yes	47 (71.2)	-0.14±0.11	0.476[a]
		No	19 (28.8)	-0.01±0.07	
Diabetes Complications	IHD	Yes	32 (48.5)	-0.26±0.10	0.375[a]
		No	34 (51.5)	-0.17±0.13	
	CHF	Yes	17 (25.8)	-0.13±0.22	0.835[a]
		No	49 (74.2)	-0.09±0.08	
	PVD	Yes	23 (34.8)	-0.08±0.08	0.825[a]
		No	43 (65.2)	-0.12±0.12	
	Diabetic ulcer	Yes	15 (22.7)	-0.11±0.12	0.945[d]
		No	51 (77.3)	-0.10±0.10	
	Amputation	Yes	3 (4.5)	-0.54±0.56	0.253[d]
		No	63 (95.5)	-0.08±0.08	

	Nephropathy	Yes	32 (48.5)	-0.08±0.13	0.468[a]
		No	34 (51.5)	-0.12±0.11	
	CKD	Yes	23 (34.8)	-0.07±0.16	0.747[a]
		No	43 (65.2)	-0.12±0.10	
	Dialysis	Yes	6 (9.1)	-0.03±0.07	0.774[d]
		No	60 (90.9)	-0.11±0.09	
	Neuropathy	Yes	28 (42.4)	-0.17±0.11	0.468[a]
		No	38 (57.6)	-0.05±0.12	
	Neurovascular disease	Yes	14 (21.2)	-0.17±0.15	0.665[a]
		No	52 (78.8)	-0.08±0.10	
Medications	Insulin	Yes	49 (74.2)	-0.09±0.09	0.873[a]
		No	17 (25.8)	-0.12±0.18	
	Glimepiride	Yes	14 (21.2)	-0.07±0.15	0.821[a]
		No	52 (78.8)	-0.11±0.10	
	Alpha-glucosidase	Yes	1 (1.5)	0.10	0.762[d]
		No	65 (98.5)	-0.11±0.08	
	Thiazolidinedione	Yes	1 (1.5)	-1.1	0.142[d]
		No	65 (98.5)	-0.09±0.08	
	SGLT2 inhibitor		23 (34.8)	-0.05±0.11	0.627[a]
	Yes		43 (65.2)	-0.13±0.11	
		No	39 (59.1)	-0.07±0.10	0.611[a]
	Aspirin	Yes	27 (40.9)	-0.15±0.14	
		No	6 (9.1)	-0.08±0.05	0.498[d]
	Ezetimibe	Yes	60 (90.9)	-0.12±0.08	
		No	51 (77.3)	-0.11±0.10	0.829[a]
	Statins	Yes	15 (22.7)	-0.07±0.12	
		No	13 (19.7)	0.07±0.11	0.308[d]
	Anticoagulation	Yes	53 (80.3)	-0.14±0.10	
		No	36 (54.5)	-0.10±0.12	0.982[a]
	Beta-blockers	Yes	30 (45.5)	-0.10±0.11	
		No	27 (40.9)	-0.14±0.16	0.684[a]
	Diuretics	Yes	39 (59.1)	-0.07±0.09	
		No			
Ocular Conditions	Retinal detachment	Yes	8 (12.1)	-0.27±0.43	0.667[e]
		No	58 (87.9)	-0.08±0.08	
	Vitreous hemorrhage	Yes	22 (33.3)	-0.27±0.21	0.263[a]
		No	44 (66.7)	-0.02±0.07	
	Glaucoma	Yes	15 (22.7)	-0.35±0.24	0.214[a]
		No	51 (77.3)	-0.03±0.08	
	Pseudophakia	Yes	37 (36.1)	-0.29±0.12	0.325[a]
		No	29 (43.9)	-0.19±0.11	
	Retinal atrophy	Yes	2 (3.0)	0.29±0.16	0.405[d]
		No	64 (97.0)	-0.11±0.08	
	Epiretinal membrane	Yes	11 (16.7)	-0.08±0.12	0.915[a]
		No	55 (83.3)	-0.11±0.10	
OCT Findings	Microaneurysms	Yes	64 (97.0)	-0.10±0.09	0.996[d]
		No	2 (3.0)	0.12±0.10	
	Exudates	Yes	7 (10.6)	0.08±0.23	0.457[d]
		No	59 (89.4)	-0.12±0.09	
	CST in microns		66 (100)	0.085	0.495[f]

	IRF	Yes	47 (71.2)	-0.16±0.10	0.290[a]
		No	19 (28.8)	0.04±0.16	
	SRF	Yes	3 (4.5)	0.04±0.05	0.707[d]
		No	63 (95.5)	-0.11±0.09	
	HRF	Yes	15 (22.7)	0.00±0.17	0.524[a]
		No	51 (77.3)	-0.13±0.09	

Abbreviations: GLP1-RA, glucagon-like peptide-1 receptor agonist; T2DM, type 2 diabetes mellitus; DR, diabetic retinopathy; BMI, body mass index; HbA1c, hemoglobin A1c; logMAR, logarithm of the minimum angle of resolution; IVIs, intravitreal injections; IHD, ischemic heart disease; CHF, congestive heart failure; PVD, peripheral vascular disease; CKD, chronic kidney disease; OCT, optical coherence tomography; IRF, intraretinal fluid; SRF, subretinal fluid; HRF, hyperreflective retinal foci.

Table S7: Change in visual acuity and change in CST per incidence of ocular surgeries

			Visual Acuity Change (LogMAR)		CST Change (microns)	
		N (%)	Mean±SD	*p*-value	Mean±SD	*p*-value
**Vitrectomy**						
At Baseline	Yes	9 (13.6)	-0.31±1.01	0.511[a]	13.1±37.3	0.227[a]
	No	57 (86.4)	-0.07±0.60		-26.7±96.3	
During Follow-up (all):	Yes	7 (10.6)	-0.28±0.66	0.457[a]	-71.3±158.5	0.391[a]
	No	59 (89.4)	-0.08±0.67		-15.4±80.1	
First Time	Yes	5 (8.8)	0.04±0.16	0.623[d]	-92.2±188.7	0.416[d]
	No	52 (91.2)	-0.11±0.69		-15.5±78.8	
Recurrence	Yes	2 (22.2)	-1.09±0.85	0.033[d]	-19.0±28.3	0.971[d]
	No	7 (77.8)	-0.71±0.65		-21.4±92.7	
**Laser therapy**						
At Baseline:	Yes	33 (50.0)	-0.25±0.76	0.078[a]	-24.2±68.3	0.802[a]
	No	33 (50.0)	0.04±0.54		-18.5±110.8	
During Follow-up (all):	Yes	24 (72.7)	-0.19±0.89	0.466[a]	-19.2±99.2	0.889[a]
	No	9 (27.3)	-0.05±0.51		-22.5±87.8	
First Time	Yes	9 (27.3)	0.13±0.86	0.267[a]	-33.0±145.9	0.683[a]
	No	24 (72.7)	-0.14±0.63		-19.5±81.4	
Recurrence	Yes	15 (45.5)	-0.39±0.87	0.139[a]	-10.9±61.9	0.621[a]
	No	18 (54.5)	-0.02±0.58		-24.4±98.7	

Abbreviations: logMAR, logarithm of the minimum angle of resolution; CST, central subfield thickness.
